# Latent psychological resilience profiles and their association with physical resilience in older patients after robot-assisted radical prostatectomy: a cross-sectional study

**DOI:** 10.3389/fonc.2026.1917474

**Published:** 2026-10-08

**Authors:** Rong Liu, Xiang Xi, Xiao Ma, Yu Zhao, Hui Zhuo

**Affiliations:** Urology Department, Chengdu Third People’s Hospital, Chengdu, China

**Keywords:** cross-sectional study, latent profile analysis, physical resilience, prostate cancer, psychological resilience

## Abstract

**Objective:**

To identify latent psychological resilience profiles in older patients after robot-assisted radical prostatectomy (RARP) and examine their association with physical resilience.

**Methods:**

A total of 300 patients with prostate cancer were recruited from a Class III Grade A hospital in Sichuan Province between December 2025 and May 2026. Assessments were conducted on postoperative day 3 using the general information questionnaire, Connor-Davidson Resilience Scale, and Physical Resilience Instrument for Older Adults. Latent profile analysis was performed using the total score on the Connor-Davidson Resilience Scale. Generalized linear regression was used to analyze the relationship between psychological and physical resilience across categories among elderly patients after RARP.

**Results:**

Three latent psychological resilience profiles were identified among older patients after RARP. Physical resilience scores differed among people with different psychological resilience. The results of generalized regression analysis showed that patients in different psychological resilience profiles of RARP patients were related to their physical resilience (*P* < 0.05).

**Conclusions:**

Older patients after RARP showed heterogeneous psychological resilience profiles, and profile membership was associated with physical resilience. Longitudinal and intervention studies are needed to determine the temporal direction and clinical utility of these profiles.

## Introduction

Prostate cancer (PC) is a major global health concern and one of the most commonly diagnosed malignancies in men (1, 2). Robot-assisted radical prostatectomy (RARP) has become an established treatment for localized PC and may reduce perioperative blood loss, postoperative pain, and length of hospital stay compared with open surgery (3). Nevertheless, patients may experience urinary, sexual, and psychological difficulties after surgery. These challenges can affect adaptation during the early postoperative period and highlight the need to understand both psychological and physical resilience (4).

Psychological resilience refers to the capacity to adapt to acute or chronic stress, adversity, and illness-related burden (5). Greater resilience has been associated with lower emotional distress and better adjustment among patients with cancer (6, 7). Physical resilience refers to the capacity to resist or recover from functional decline after a health stressor, such as surgery, acute illness, or injury (8). Although conceptually distinct, psychological and physical resilience may be interrelated during postoperative adaptation; however, their temporal relationship in older patients after RARP remains unclear.

Few studies have examined heterogeneity in psychological resilience or its relationship with physical resilience among older patients after RARP. Latent profile analysis is a person-centered method that can identify subgroups with distinct patterns across continuous indicators (9, 10). Variable-centered analyses estimate average associations and may obscure whether patients show different configurations across multiple resilience dimensions. A person-centered analysis can explore such heterogeneity by identifying groups with similar indicator patterns. However, whether these patterns provide information beyond the continuous resilience score is an empirical question and was treated as exploratory in this study. Accordingly, this cross-sectional study aimed to identify latent psychological resilience profiles and examine their association with physical resilience. We hypothesized that physical-resilience scores would differ across the identified profiles.

## Methods

### Subjects

We planned to recruit 300 patients; the final sample comprised 250 participants. Taken together, the balanced profile sizes, class separation, model-fit indices, interpretability, and parsimony suggest that the sample of 250 participants was reasonably adequate for an exploratory three-profile solution. However, no Monte Carlo simulation-based sample-size or power analysis was performed. Therefore, the sample size should not be described as formally established as sufficient, and the stability and generalizability of the profile solution require confirmation in larger, multicenter samples.

Inclusion criteria: ①age 60-85; ②willing to receive RARP (11);③ability to communicate and willingness to participate in the study. Exclusion criteria:①severe infection;②severe cognitive impairment or mental illness;③severe organ failure and loss of consciousness;④refusal to participate in the study or concomitant participation in other treatment modalities. The Ethics Committee of the Third People’s Hospital of Chengdu approved this study (2026-S-32). Written informed consent was obtained from the patients, and relevant ethical standards were met.

### Research tools

#### General information questionnaire

Sociodemographic and clinical characteristics were collected using a study-specific questionnaire. Patient-reported variables included age, marital status, education level, occupation, place of residence, living arrangement, and monthly household income per capita. Body mass index and pathological stage were obtained from the electronic medical record and verified by the research team.

#### Connor-Davidson Resilience Scale

The CD-RISC was developed by Connor et al. in 2003 (12).e Chinese version was revised by Yu et al. in 2007. It comprises 25 items across three dimensions: optimism, self-reliance, and tenacity (13). The 5-point Likert scale (0-4) was used; positive scores were assigned; the range was 0-100, with higher scores indicating better psychological resilience in patients. The Cronbach α coefficient of the scale was 0.886. The results of this study show that the Cronbach α coefficient of this scale is 0.955. Psychological resilience was assessed on postoperative day 3.

#### Physical Resilience Instrument for Older Adults

Developed by Hu et al. (14), Physical resilience was assessed using the 16-item Physical Resilience Instrument for Older Adults, which evaluates positive thinking, coping and lifestyle adjustment, and belief and hope in response to health stressors. The instrument was translated into Chinese by Li Jiaxin et al. (15).It includes 3 dimensions of positive thinking; coping and lifestyle adjustment; and belief and hope, with a total of 16 items. The scale is scored on a 5-point Likert scale from “strongly disagree” to “strongly agree” (1 to 5), with total scores ranging from 16 to 80, with higher scores indicating greater physical resilience. The Cronbach α coefficient of the scale was 0.886. Internal consistency in the present sample was acceptable but modest (Cronbach α = 0.711). Physical resilience was assessed on postoperative day 3.

### Quality control

Two trained graduate researchers administered the questionnaires using standardized instructions and collected them immediately after completion. Participants could request clarification of item wording; researchers did not suggest responses. When a participant was unable to complete the questionnaire independently, a trained researcher read each item verbatim and recorded the participant’s answer. Two researchers independently checked questionnaire completeness and data entry, and discrepancies were resolved against the source questionnaire. Questionnaires with missing data were excluded.

### Statistical analysis methods

Normally distributed continuous variables were summarized as means ± standard deviations. Categorical variables were summarized as frequencies, and the chi-square test or Fisher’s exact test was used. Analysis of variance and *post hoc* tests were used to test the differences in physical resilience among the latent profiles of psychological resilience. Generalized linear regression analysis was used to explore the relationship between latent categories of psychological resilience and physical resilience, and *P* < 0.05 was considered statistically significant. Latent profile model construction was fitted using Mplus 8.3. The model-fit indices mainly included Akaike Information Criterion(AIC) and Bayesian Information Criterion (BIC), Adjusted Bayesian Information Criterion (aBIC), and the Luo-Mendel-Rubin corrected likelihood ratio test(LMRT), bootstrap-based likelihood ratio test (BLRT) and Entropy, where smaller values of AIC, BIC and aBIC indicated better model fit. LMRT compares models across latent classes, and P<0.05 indicates that the model with K classes is better than the model with K−1 classes (16). When Entropy≥0.8, the model’s classification accuracy is≥90% (17).

## Results

### General information

Of the 300 patients initially considered, 30 patients refused surgical treatment for fear of surgical complications, 10 patients were transferred to other hospitals before surgical treatment, 5 patients were transferred to the Intensive Care Unit for further treatment due to their serious underlying diseases, and 5 patients were also receiving other treatment after surgery. These patients were excluded from the study. A total of 250 patients participated in this study, with a mean age of 66.26 ± 5.479 years. Marital status: 210 cases were married, 6 cases were unmarried, 21 cases were widowed, and 13 cases were divorced. Education level: 43 cases had junior college or above, 97 cases had junior high school or senior high school, and 110 cases had primary school or below. Occupation: 202 cases were retired and 48 cases were unemployed. 106 cases lived in urban areas and 144 cases lived in rural areas. Living arrangement: 30 cases lived alone, 203 cases lived with their spouse/children, and 17 cases lived with other relatives. Per capita monthly income: 73 cases of 5000 and above, 119 cases of 3000-5000, 45 cases of 1000-3000, 13 cases of 1000 and below; Pathological stage: 34 cases of stage 1, 138 cases of stage 2, 108 cases of stage 3.

### Latent profile analysis of psychological resilience in elderly patients after RARP

Latent profile models with one to five profiles were fitted to conduct an exploratory latent profile analysis of psychological resilience (total score of CD-RISC) in elderly patients after RARP. AIC, BIC, and aBIC decreased as the number of profiles increased. However, LMRT and BLRT were not significant for the four- and five-profile models, and Entropy values were relatively large for the two-profile model. For the three-profile model, the decreasing trend of AIC, BIC and aBIC became less pronounced. LMRT and BLRT both reached significant levels (*P* < 0.05). Therefore, after comprehensive consideration, the three-profile model was considered for describing the psychological resilience grading of elderly patients after RARP. The attribution probabilities of different resilience scales were 96.4%, 98.6% and 98.8%, respectively, which showed high reliability. See **Table 1** for details.

**Table 1:** Latent profile analysis of psychological resilience in elderly patients after RARP.

Categories	AIC	BIC	aBIC	Entropy	LMP-LRT(*P*)	BLRT(*P*)	Class probability
1	18895.044	19071.117	18912.613				
2	16836.140	17103.771	16862.845	0.963	<0.001	<0.001	0.48/0.51
3	16420.716	16779.905	16456.556	0.947	0.033	0.034	0.32/0.32/0.36
4	15669.696	16120.443	15714.672	1.000	0.718	0.719	0.29/0.37/0.04/0.29
5	15330.388	15872.693	15384.500	0.984	0.089	0.091	0.29/0.29/0.18/0.04/0.19

### Potential profile characteristics and naming of psychological resilience in elderly patients after RARP

**Figure 1** presents the cluster-specific estimated probabilities of resilience for the LPA three-class model, based on resilience scores among elderly patients after RARP. Based on Kumpfer’s point that psychological resilience is influenced by internal and external factors, three possible adaptation outcomes are identified: resilience reorganization (psychological resilience increases), dynamic balance reorganization (psychological resilience returns to the pre-stress level), and maladaptive reorganization (psychological resilience decreases) (18). Therefore, the C1 group had a high overall score for psychological resilience (85.21 ± 8.74), high scores for tenacity, self-improvement, and optimism, and was characterized by faith and hope. This group was labeled high psychological resilience—positive belief and hope and accounted for 32% of participants. Patients in C2 had an intermediate psychological resilience score (58.03 ± 4.92), and they could complete certain responses and adjustments in the face of pressure and challenge. This group was labeled the medium psychological resilience group—limited coping adjustment and accounted for 32% of participants. The C3 group had a low score for psychological resilience (43.89 ± 6.69). This group was characterized by pessimistic thinking and was classified as the low psychological resilience group—negative thinking and emotions, accounting for 36%.

**Figure 1:**
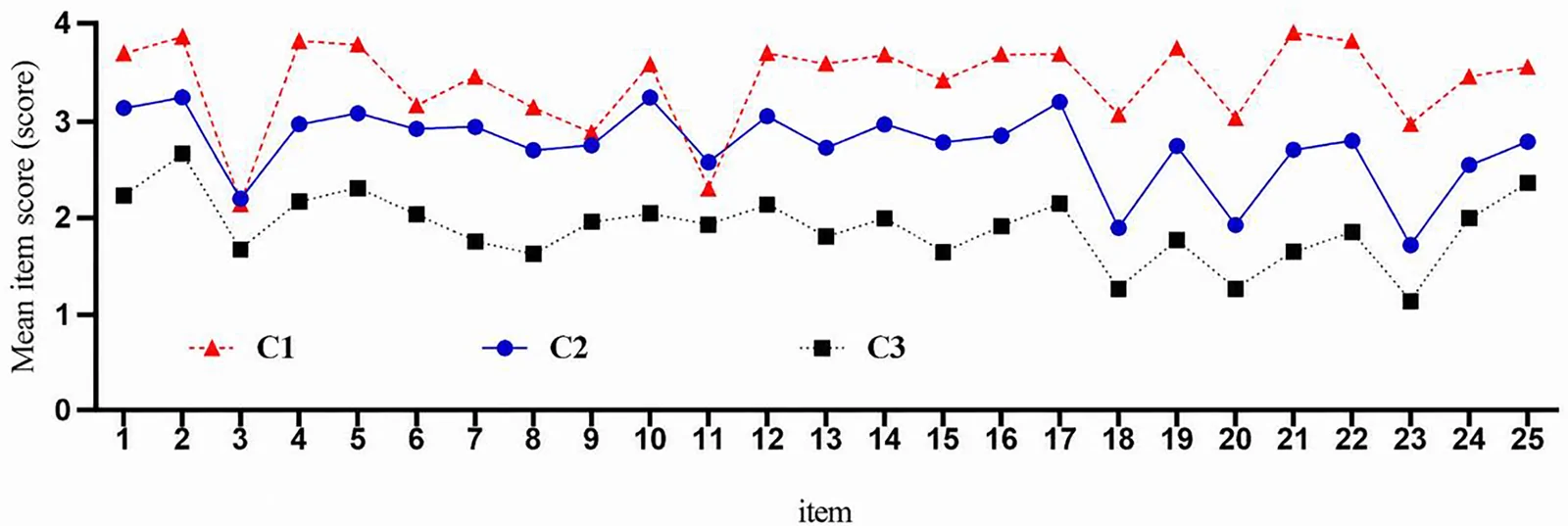
Estimated indicator means across the three latent psychological resilience profiles. Lines represent the profile-specific means of Connor-Davidson Resilience Scale. C1, C2, and C3 denote the three retained profiles.

### Potential profile analysis of psychological resilience of elderly patients after RARP with different characteristics

**Table 2** shows no statistically significant differences in the distribution of the three psychological resilience profiles among elderly patients after RARP, regardless of age, BMI, or education level (*P*>0.05). There were significant differences in occupation, residence, per capita monthly income, living arrangement, marital status, and pathological stage (*P* < 0.05).

**Table 2:** Differences in psychological resilience profile distribution of elderly patients after RARP with different characteristics.

item	C1 (n=80)		C2 (n=80)	C3 (n=90)	Test statistic	*P*
**Age**	66.58 ± 5.80	65.66 ± 5.39		67.54 ± 5.80	1.23	0.293
**BMI**	24.80 ± 1.38	25.23 ± 1.35		24.45 ± 1.49	0.17	0.847
Educational level					4.68	0.321
Primary school and below	34	33		50		
Middle School and High School	29	32		27		
College or above	17	15		13		
Occupations					18.75	<0.001
Retired	73	69		60		
Unemployed	7	11		30		
Place of residence					17.71	<0.001
Rural areas	48	46		28		
Cities	32	34		62		
Marital status					34.23	<0.001
Married	76	73		61		
Unmarried	2	2		2		
Divorced	0	1		12		
Widowed	2	4		15		
Monthly income per capita					44.55	<0.001
<1000	1	3		9		
1000~3000	7	7		31		
3000~5000	35	48		36		
>5000	37	22		14		
Living arrangement					17.16	0.002
Live alone	8	3		17		
Living with a spouse/children	63	75		70		
Living with other relatives and friends	9	2		3		
Pathological staging					4.147	0.017
Stage 1	3	1		0		
Stage 2	45	43		50		
Stage 3	32	36		40		

### Physical resilience of elderly RARP patients with different psychological resilience

The results of the single-factor method showed that Physical resilience scores differed across the three psychological resilience profiles in each of the three domains(*P* < 0.05). The results of the LSD test showed that in the three dimensions of physical resilience, the scores of patients in group C1 were higher than those in group C2 and group C3, and these differences were statistically significant(*P* < 0.05). See **Table 3** for details.

**Table 3:** Physical resilience of different psychological resilience profiles.

Psychological resilience profile	Positive thinking	Coping and lifestyle adjustment	Belief and hope	Total score	LSD
C1	13.46 ± 1.28	20.14 ± 1.57	20.56 ± 1.62	54.16 ± 2.63	1>2>3
C2	11.99 ± 1.31	18.16 ± 1.27	18.23 ± 1.81	47.80 ± 3.29	1>2>3
C3	10.63 ± 1.32	16.64 ± 1.37	16.67 ± 1.50	43.46 ± 2.61	1>2>3
*F*	99.74	130.73	119.67	47.007	
*P*	<0.001	<0.001	<0.001	<0.001	

C1 = 1, C2 = 2, C3 = 3.

### Association between psychological resilience profiles and physical resilience in older patients after RARP

We used sociodemographic and clinical characteristics and latent profiles of psychological resilience in elderly patients after RARP as independent variables, and the Physical Resilience Instrument for Older Adults score as the dependent variable in generalized linear regression analysis. **Table 4** shows the assignment of independent variables, and **Table 5** presents the results of the generalized linear regression analysis.

**Table 4:** Assignment of independent variables.

Item	Assignment method
Age	Original value
BMI	Original value
Occupation	Retired =1, unemployed =2
Educational level	Primary school and below =1, junior high school and senior high school =2, junior college and above =3
Marital status	Married =1, unmarried =2, widowed =3, divorced =4
Place of residence	Town =1, rural =2
Living arrangement	Living alone =1, living with a pouse/children =2, living with relatives and friends =3
Per capita monthly income	5000 and above =1, 3000-5000 = 2, 1000-3000 = 3,1000 and below =4
Pathological staging	Stage 1 = 1, stage 2 = 2, stage 3 = 3
Psychological resilience profiles	C3 = 1, C2 = 2, C1 = 3

**Table 5:** Generalized linear regression analysis of different psychological resilience profiles and physical resilience in elderly patients after RARP (n=250).

Variables of interest	β	S.E.	Wald X^2^	95%CI	*P*
C1	10.707	0.44	605.09	9.85-11.56	<0.001
C2	4.344	0.44	99.62	3.49-5.20	<0.001

C3 was used as a reference.

## Discussion

Psychological resilience and physical resilience are conceptually related but not interchangeable. In this study, the CD-RISC assessed perceived psychological resources for adaptation to adversity, whereas the PRIFOR assessed perceived capacity to withstand or recover from a physical health stressor. Nevertheless, PRIFOR domains such as positive thinking, coping and lifestyle adjustment, and belief and hope overlap with psychological content assessed by the CD-RISC. The observed association may therefore partly reflect shared construct content and concurrent self-report measurement, rather than an association with objective postoperative recovery alone. In this sample, there were also differences across sociodemographic and clinical characteristics. However, these comparisons do not identify any single feature that produces a particular profile. The present study identified three latent psychological resilience profiles in elderly patients after RARP, indicating heterogeneity in physical resilience-related characteristics. The physical resilience score decreased from C1(54.16 ± 2.63) to C2(47.80 ± 3.29) and C3(43.46 ± 2.61). These concurrent differences should be interpreted as associations rather than evidence that resilience determines postoperative recovery. Elderly patients undergoing RARP may face functional changes as well as uncertainty about urinary control, sexual function, recurrence, and resumption of daily activities (19, 20). Studies have shown that low physical resilience is associated with unmarried patients with limited social support, unemployed patients, and patients with lower socioeconomic status, and that patients with higher risk of psychological distress can be identified early (21). In the future, it is necessary to pay more attention to the postoperative physiological and psychosocial needs of patients, strengthen their psychological resilience, and help patients cope with postoperative challenges.

This study identified three psychological resilience profiles:C1 group (high psychological resilience—positive belief and hope), C2 group (medium psychological resilience group—limited coping adjustment) and C3 group (low psychological resilience group—negative thinking and emotions). The psychological resilience score of patients in the C1 group was significantly higher than that of patients in the C2 and C3 groups. **Table 2** shows that the three potential patient categories differ in occupational status, residence, marital status, per capita monthly family income, lifestyle, and pathological stage (*P* < 0.05). Therefore, it is recommended in the future that the focus of clinical care for patients after RARP should be adjusted according to their different sociodemographic and clinical characteristics. Patients in group C1 have the best psychological adaptation after RARP, but there may be problems such as high expectations for disease prognosis and postoperative quality of life, and an incomplete understanding of the disease. In the future, we can focus on prostate cancer health management and recurrence prevention resources to support them in maintaining a healthy lifestyle, meet their higher-level information and social needs, and improve their quality of life. Patients in group C2 may benefit from timely support, which gives us certain enlightenment. In the future, we can provide targeted postoperative rehabilitation training and positive feedback, such as postoperative self-care, access to disease information, and sharing examples of successful recovery (22), to enhance self-care ability and other skills and promote their transition to group C1. Patients in group C3 are in a fragile state and needed high-intensity support and basic nursing care (23). Future nursing care could focus on establishing patient support groups and encouraging family involvement in care. Health care workers could help patients identify and address negative thinking patterns, such as catastrophizing and all-or-nothing thinking. They could also guide patients toward a realistic understanding of the disease and recovery after RARP. Additional support could help patients develop basic postoperative coping skills and rebuild their sense of control and confidence (24).

Ecological systems theory suggests that inadequate preparation for postoperative changes in physical function and the social environment may affect their postoperative physical recovery (25).From a clinical perspective, the findings support considering psychological and physical resilience together during early postoperative assessment. They do not yet justify assigning patients to fixed risk groups or implementing profile-specific interventions. One possible explanation is that psychological and environmental resources may be related to symptom assessment, coping behaviors, treatment engagement, and maintenance of daily activities, which in turn may be related to perceived physical resilience (26–28). Neuroendocrine and immune pathways have also been implicated as potential links between psychological stress and physical recovery (29, 30).A more defensible implication is that patients reporting lower psychological resilience may warrant further assessment of physical recovery needs, coping resources, and available support. However, these explanations remain hypothetical, since coping behavior, treatment adherence, inflammatory or neuroendocrine biomarkers, and longitudinal recovery trajectories were not assessed. Reverse causality is equally plausible: patients with better physical recovery may report greater confidence, hope, and resilience. Future multicenter longitudinal studies with repeated assessments, objective measures of recovery, and prespecified intervention testing are needed to determine whether these characteristics are stable, modifiable, and clinically relevant.

## Conclusion

Based on the latent profile analysis method, this study identified 3 potential categories of resilience in elderly patients after RARP: group C1 (high resilience - positive beliefs and hope), group C2 (medium resilience - limited coping adjustment), and group C3 (low resilience - negative thoughts and emotions). After RARP, elderly patients in different resilience categories differed significantly in demographic and clinical characteristics. These findings may inform nursing support for psychological adaptation and physical recovery, and provides new ideas and directions for nursing psychological adaptation and physical recovery ability in elderly patients after RARP to improve their quality of life.

The identified profiles were primarily ordered by overall resilience level, and the present study does not establish that they represent qualitatively distinct adaptation patterns or provide greater clinical utility than the continuous CD-RISC score. The person-centered analysis should therefore be regarded as exploratory. Its incremental value would require replication of non-parallel multidimensional patterns, stable classification, external validation, and evidence that profile membership adds clinically relevant information beyond conventional score-based assessment. In this cross-sectional sample, psychological resilience correlated with physical resilience scores. Because both constructs were measured simultaneously, the direction of the association could not be determined. Reverse causality, residual confounding, and common method variance may have influenced the observed relationships. The cross-sectional design of this study cannot reveal the dynamic causal relationship between psychological and physical resilience over time in elderly patients after RARP. Future studies could explore the dynamic trajectory of the interaction using a longitudinal design with repeated measurements.

## Data Availability

The original contributions presented in the study are included in the article/supplementary material. Further inquiries can be directed to the corresponding author.
